# Decoding Semi-Constrained Brain Activity from fMRI Using Support Vector Machines and Gaussian Processes

**DOI:** 10.1371/journal.pone.0035860

**Published:** 2012-04-26

**Authors:** Jessica Schrouff, Caroline Kussé, Louis Wehenkel, Pierre Maquet, Christophe Phillips

**Affiliations:** 1 Cyclotron Research Centre, University of Liège, Liège, Belgium; 2 Department of Electrical Engineering and Computer Science, University of Liège, Liège, Belgium; 3 Giga-R, Systems Biology and Chemical Biology, University of Liège, Liège, Belgium; 4 Department of Neurology, Liège University Hospital, Liège, Belgium; Indiana University, United States of America

## Abstract

Predicting a particular cognitive state from a specific pattern of fMRI voxel values is still a methodological challenge. Decoding brain activity is usually performed in highly controlled experimental paradigms characterized by a series of distinct states induced by a temporally constrained experimental design. In more realistic conditions, the number, sequence and duration of mental states are unpredictably generated by the individual, resulting in complex and imbalanced fMRI data sets. This study tests the classification of brain activity, acquired on 16 volunteers using fMRI, during mental imagery, a condition in which the number and duration of mental events were not externally imposed but self-generated. To deal with these issues, two classification techniques were considered (Support Vector Machines, SVM, and Gaussian Processes, GP), as well as different feature extraction methods (General Linear Model, GLM and SVM). These techniques were combined in order to identify the procedures leading to the highest accuracy measures. Our results showed that 12 data sets out of 16 could be significantly modeled by either SVM or GP. Model accuracies tended to be related to the degree of imbalance between classes and to task performance of the volunteers. We also conclude that the GP technique tends to be more robust than SVM to model unbalanced data sets.

## Introduction

Two of the most fundamental questions in the field of neurosciences are how information is represented in different brain structures, and how this representation evolves over time. Functional Magnetic Resonance Imaging (fMRI) is a powerful tool to record brain activity and allows investigating these questions. Functional MRI can map brain activity with a spatial resolution of a few cubic millimeters and a typical temporal resolution in the order of 1 or 2 seconds. Until recently, the methods used to analyze such data focused on characterizing the individual relationship between a cognitive or perceptual state and each voxel, i.e. following a univariate approach. A well-known univariate technique is Statistical Parametric Mapping [1], detecting which voxels show a statistically significant response to the experimental conditions. However, there are limitations on what can be learned about the representation of information by examining voxels in a univariate fashion. For instance, sets of voxels considered as non-significant by the SPM analysis of one experimental condition might still carry information about the presence or absence of that condition if considered altogether.

Multivariate methods such as Multi-Voxel Pattern Analyses (MVPA) allow an increased sensitivity to detect the presence of a particular mental representation. These multivariate methods, also known as brain decoding or mind reading, aim at associating a particular cognitive, behavioral or perceptual state to specific patterns of regional brain activity [2], [3]. During the last years, methods such as Support Vector Machines (SVM, [4]), Linear Discriminant Analysis [5] or Gaussian Naïve Bayes classifiers [6] were applied to fMRI times series to predict, from individual brain activity, the patterns of perceived objects [7]–[10], mental states related to memory retrieval [11], [12] or even hidden intentions [13]. Gaussian Processes classifiers (GP, [14]), which provide a principled probabilistic approach to kernel machine learning, have been recently developed to allow for classifying more difficult data sets, such as predicting subjective pain intensity [15]. In most of these studies, the experimental design completely controlled the nature, timing and duration of experimental trials, and temporally isolated experimental conditions from one another. However, a more realistic situation consists of simultaneous or rapidly succeeding mental states. In order to improve brain decoding in these adverse conditions, we tested and estimated the accuracy of various classification schemes (namely SVM and GP combined with feature extraction methods) on two fMRI sessions. In a first well-controlled session, participants viewed a regularly paced series of images of three classes displayed at specific screen positions according to a pre-specified two-dimensional path. In a second session, participants had to mentally retrieve short sections of this path. The latter session implied uneven numbers of short events with possibly overlapping patterns of brain activity, leading to a more complex classification problem.

The aim of this manuscript was thus to decode self-paced and possibly overlapping multiclass events. Since this degree of freedom was not achieved in the previous designs of decoding experiments, we assessed the respective performance of several classification procedures, as classification accuracy may depend heavily on the data set [16].

## Materials and Methods

### Population

A group of 16 volunteers (8 females), aged between 19 and 29 years (mean 24.44), participated in the study. This study was approved by the Ethical Committee of the Faculty of Medicine of the University of Liège. All subjects were fully informed, gave their written informed consent and were paid for their participation. All included participants were non-smoking, healthy right-handed students. The volunteers were screened for anxiety (Beck anxiety inventory, [17]), depression (Beck depression inventory II, [18]), sleep quality (Pittsburgh sleep quality index, [19]), chronotype (Horne and Ostberg morningness-eveningness questionnaire, [20]), excessive daytime sleepiness (Epworth sleepiness scale, [21]), laterality (Edinburgh Inventory, [22]), amount and content of daydreams (Imaginal Process Inventory- http://www.themeasurementgroup.com/evaluationtools/ipi.htm). The subjects presented no medical, traumatic, psychiatric or sleep disorders. During the 7 days preceding the experiment, volunteers followed a regular sleep schedule, verified by wrist actigraphy and sleep diaries.

### Experimental design

**Figure 1:**
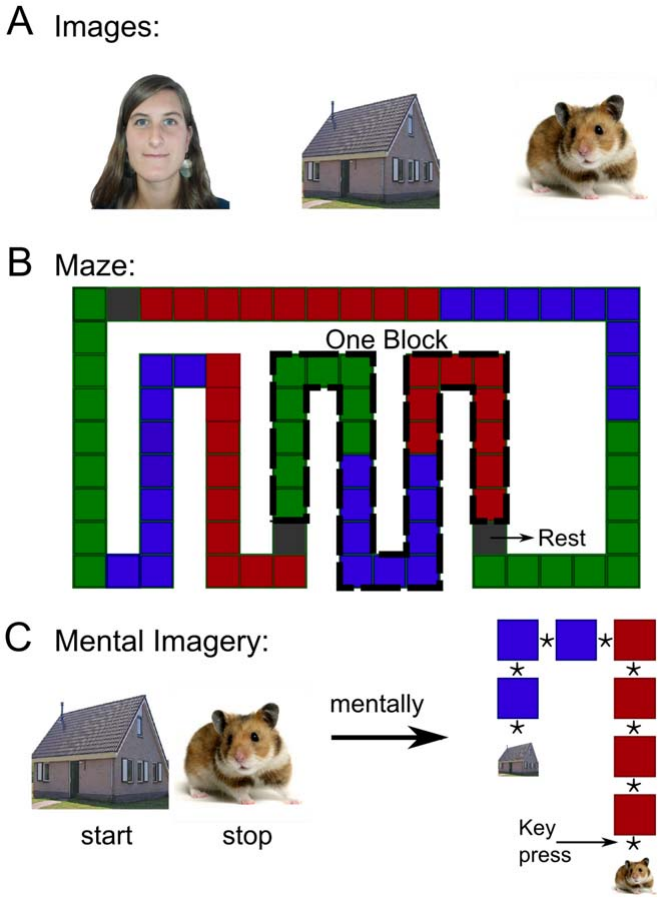
Illustration of the experimental setup for the two sessions considered further for modeling. A Example of images of faces, buildings and animals presented to the subject. In total, 81 different images were used. B Synoptic view of the maze, green areas stand for images of faces, blue areas, buildings and red areas, animals. The succession of three areas of each color is called a block. C A mental path begins with the start and stop points being displayed on the screen. The subject then travels mentally in the maze, mentally visualizing all the images comprised in the path and pressing a key every time he visualizes the required image.

All volunteers underwent three successive fMRI sessions. During the first session, further referred to as the functional ‘localizer’, images of faces, buildings and animals were presented in random order at the centre of the screen during 500 ms with an inter-stimulus interval of 1500 ms (Figure 1.A). The purpose of this session was both to identify brain areas responding specifically to the three image types and to eschew novelty effects during subsequent sessions. During the second session, referred to as ‘exploration’, the same images were displayed one at a time for 3 seconds, each image being assigned a specific location on the screen. The order of presentation followed a predefined sequence of contiguous screen positions in such a way that volunteers had the impression of following a path throughout a bidimensional maze (Figure 1.B). The complete maze consisted of three blocks of 27 consecutive images within which the 3 categories of images were always presented in the same order (i.e. 9 faces, 9 buildings and 9 animals). Between blocks, a fixation cross was displayed for 15–18 seconds. To ensure optimal encoding, the whole path was repeated five times during the scanning session. Volunteers were instructed to pay attention to each image, to their location on the screen and to their succession. During the third session, further referred to as ‘mental imagery’, volunteers were presented with 54 memory tests. During each test, two images, simultaneously displayed on the screen for 4 seconds, represented the starting and target positions of a path that the volunteers would have to follow mentally (Figure 1.C). The mental trajectories included 3 to 6 images (average 4.5) of a same category. For each image that they could conjure up during this mental travel, volunteers had to signal by a key press whether it was a face, a building or an animal (one finger and key per condition). However, subjects had the possibility to skip a path if they could not remember any part of it. The expected number of images of each type was perfectly balanced between categories.

A memory test was finally performed outside the scanner, in order to behaviorally assess the accuracy of the spatial knowledge acquired by the volunteers. They were presented with the previously seen pictures and 48 novel images in random order. For each trial, an image was displayed on the screen at a specific location and participants had to specify whether this image was part of the maze and, if they believed it was, if it was displayed at its correct location.

The classification procedures were only applied on the last two sessions, i.e. the exploration and mental imagery. These were designed such that the subject performed a totally controlled task during the exploration session, whereas during mental imagery, the pace and succession of mental representations were not constrained by external stimuli but only to the volunteer's capacity to retrieve the learned stimuli and their location. A further characteristic of the exploration session was that within a block, no rest period was introduced at the transition between images of different categories. As a consequence, fMRI signals of different classes of events were expected to overlap, making correct classification more complex. Furthermore, during the mental imagery session, the event duration was not fixed and depended entirely on the speed at which each subject recalled the requested images. This resulted in event durations varying between 200 ms and 4000 ms, with most events during less than 2000 ms.

### Data acquisition

Functional MRI time series were acquired on a 3T head-only scanner (Magnetom Allegra, Siemens Medical Solutions, Erlangen, Germany) operated with the standard transmit-receive quadrature head coil. Multislice T2*-weighted functional images were acquired with a gradient-echo echo-planar imaging sequence using axial slice orientation and covering the whole brain (34 slices, FoV = 192×192 mm^2^, voxel size 3×3×3 mm^3^, 25% interslice gap, matrix size 64×64×34, TR = 2040 ms, TE = 30 ms, FA = 90°). The three initial volumes were discarded to avoid T1 saturation effect. The static field inhomogeneities were measured using a field mapping sequence (32 slices, FoV = 220×220 mm^2^, voxel size = 3.4×3.4×3 mm^3^, 30% interslice gap, TR = 517 ms, TE = 4.92 and 7.38 ms, FA = 90°), using the same brain coverage and slice orientation as for the EPI sequence. Finally, a high-resolution T1-weighted image was acquired for each subject (3D MDEFT [23]; TR = 7.92 ms, TE = 2.4 ms, TI = 910 ms, FA = 15°, FoV = 256×224×176 mm^3^, 1 mm isotropic spatial resolution).

### Functional MRI data analysis

The classification techniques considered here are used in a within-subject and binary way, i.e. the categories were compared pairwise for each subject. Different (combinations of) techniques of feature extraction and classification were tested. These included a General Linear Model (GLM, multiple regression model that considers the voxels in a univariate way [1]), Support Vector Machines (SVM) and Gaussian Processes (GP). The selection of these methods was inspired by previous works on pattern recognition and brain decoding [5], [6] and [24].

#### Image preprocessing

The images were preprocessed using SPM8 (www.fil.ion.ucl.ac.uk/spm). First, spatial deformations induced by the field inhomogeneities were estimated using the FieldMap toolbox [25]. The images were corrected for the differences in slice acquisition time (slice timing correction to the middle slice), then were simultaneously realigned and unwarped to account for the subject movements in the scanner and for the interaction between these movements and the spatial deformations. Finally, the images were smoothed using a Gaussian function with a 4 mm FWHM kernel to reduce high spatial frequency noise in the images.

#### Signal extraction

For exploration and mental imagery, the whole time series of all voxels were extracted. A GLM was used to regress out movement effects (estimated by realignment parameters) and low frequency drifts (cutoff: 1/128 Hz). The signal corresponding to stimulus onsets was then extracted, considering a hemodynamic response function (HRF) delay of 6 seconds (according to [26]). To avoid decoding the signal linked to motor activity in the mental imagery session, the scans selected for further classification were the ones preceding the key presses (after correction for HRF delay). Overlapping events were also handled with care, preventing the inclusion of two different stimuli in the same TR. The signal was finally averaged over specific time-windows to increase the Signal-to-Noise Ratio (SNR; [9] and [27]). For the exploration session, the average was performed over the time the stimulus was presented (i.e. 3 seconds). For the mental imagery session, this average was performed over the interval between two key presses, with a maximum of 2 scans (i.e. 4.080 seconds) to avoid the inclusion of episodes of task-unrelated thoughts.

#### Feature selection

Reducing the number of features helps data understanding, reduces the memory storage requirements [28], and mitigates the effects of the “curse of dimensionality” [5], while improving overall performance [24]. The feature extraction techniques generally consist either in univariate methods such as the General Linear Model (GLM) analysis or in multivariate methods such as SVM. As these two kinds of techniques can be combined to obtain an optimal subset of variables [24], we considered both techniques separately then in combination.

The univariate feature selection chosen in the present work was a GLM analysis. This technique seemed the most straightforward, as it is the most common approach to analyze fMRI data in an event-related experimental design, and was already proven a useful feature selection method in previous works [6], [10], [27]. Using a GLM analysis, the subset of ‘active’ voxels (i.e. whose activity was statistically significantly correlated with the three conditions) was determined [6]. From the resulting F maps, two voxel sets were selected: (1) all voxels above an F-threshold of 0.5 (referred to as ‘global GLM feature selection’) and (2) the 1000 most significant voxels [10] (referred to as ‘specific GLM feature selection’). This number of 1000 was chosen as the compromise between an under-constrained space (dimensionality much larger than 1000), which might lead to overfitting, and an over-constrained space (dimensionality much smaller than 1000), which would make the second feature selection step useless.

**Figure 2:**
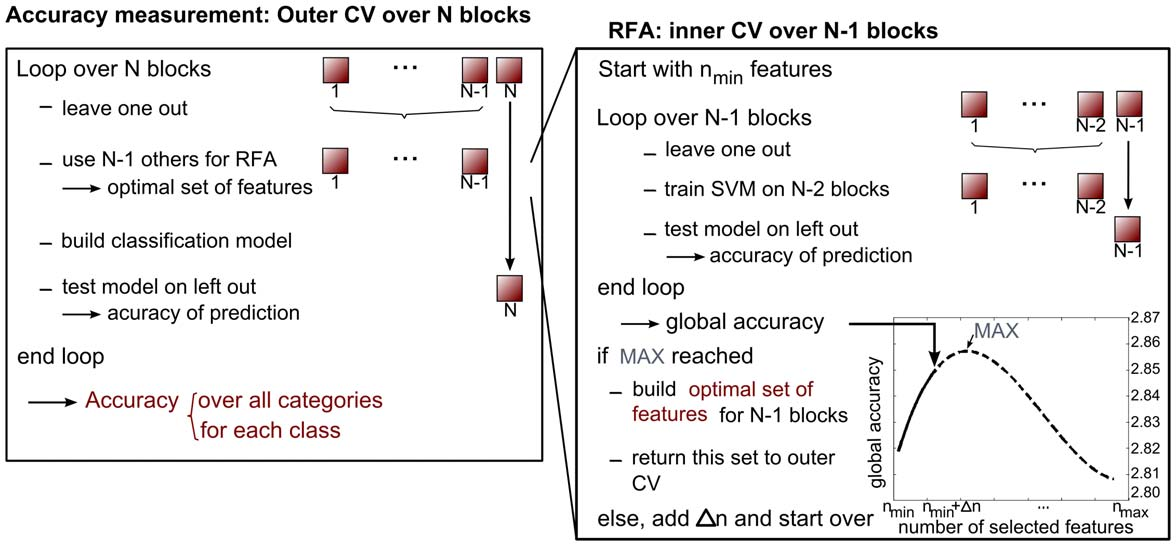
Recursive Feature Addition (RFA) process and Cross-Validations (CV) used in procedures 3 to 5. The accuracy measure is presented in the left box: from the outer CV, one block (containing m events) is left out which will be used later to test the final classification accuracy and does not enter the feature selection process. The N-1 other blocks enter the RFA process (right box) to define the optimal set of features, which will be used to build the model: the inner CV tests an SVM model built on N-2 blocks, leading to a value for the global accuracy (sum of the accuracies obtained for each binary comparison). This inner CV loop is repeated until the accuracy curve starts to decrease and hence a maximum value of global accuracy is reached, corresponding to an optimal subset of variables. N represents the number of blocks, n_min_ (respectively n_max_,) represents the minimum (respectively maximum) number of selected features and Δn, the step size.

In the multivariate procedure, a binary SVM using linear kernels was used to rank the voxels according to their ‘discriminating power’, which was computed from their specific weights [6]. The voxels with the largest absolute weights were selected for further modeling. The number of selected voxels systematically varied from 5 (per condition and binary comparison) to 150 at most, by increments of 25 (respectively n_min_, n_max_ and Δn on Figure 2). These parameters were fixed arbitrarily. At each iteration the sum of the accuracy of the three binary models on a left-out block was taken as a global accuracy measure. In general, the addition of relevant features increases the accuracy of the classification while adding irrelevant features leads to a decrease in accuracy [5]. When recursively adding features, this global accuracy measure is therefore expected to increase and then decrease (when irrelevant features are being added to the relevant ones). The set of voxels leading to the highest global accuracy (i.e. when the global accuracy starts decreasing compared to the 2 previous iterations) was then selected for the classification analysis (see Figure 2 for an illustration of the process). Features are thus added recursively following a ‘Recursive Feature Addition’ (RFA) procedure, in contrast to ‘Recursive Feature Elimination’ in which features are recursively discarded [29]. RFA can then be assimilated to a forward wrapper feature selection, with a cost function based on the global accuracy as objective.

In all cases, feature selection was performed on the training set only to ensure unbiased estimations of the accuracy with the test set (see below).

#### Classification methods

Two modeling techniques were considered to classify the data: SVM and GP. Because SVM is widely used in the field of brain decoding ([13], [27]–[30]), it was considered as the method of reference. SVM was computed using linear kernels and a fixed regularization hyperparameter C = 1 ([27], [31]).

GP was used to compute probabilities of classes, primarily with the goal of improving overall classification accuracy on mental imagery data. Furthermore, GP is presented as the generalization of different classifiers (e.g. neural networks, [14]) and seemed therefore appropriate for our particular data, in which no technique could easily be selected a priori. For GP classification, we used the Expectation Propagation approximation of the posterior mode [14], which recursively updates local parameters of the distribution. The covariance matrix was modeled by an inhomogeneous linear kernel matrix (*k*, [14]):
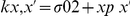
where Σ_p_ represents a general covariance matrix controlling for the precision of the prior distribution and σ_0_^2^ accounts for a bias term. In the present work, both Σ_p_ and σ_0_^2^ are expressed using only one hyperparameter σ, i.e. 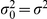 and Σ*_p_ = σ* ^2^ *I*, *I* being the identity matrix. The logarithm of σ was initialized to 1. Before training, the kernel matrix was centered, independantly for the training and test sets.

#### Classification procedures and accuracy measures

The different feature extraction, GLM and Recursive Feature Addition (RFA), and classification (SVM and GP) methods were combined in five distinct ‘procedures’ (Table 1), which were conducted as follows:

*Procedure 1:* The specific GLM feature selection method identifies the 1000 most ‘active’ voxels in our experiment. SVM binary classification is then performed for each pair of image types.
*Procedure 2:* The specific GLM feature selection method identifies the 1000 most ‘active’ voxels in our experiment. GP binary classification is then performed for each pair of image types.
*Procedure 3:* The global GLM feature selection method identifies all ‘active’ voxels above the F-threshold of 0.5. Then, RFA is performed with a range of selected features from 5 to 150 [10], selecting the number of features leading to the best generalization accuracy. GP classification is then performed using this number of selected features.
*Procedure 4:* The specific GLM feature selection method identifies the 1000 most ‘active’ voxels in our experiment. RFA is then performed with a range of selected features from 5 to 150 [10], selecting the number of features leading to the best generalization accuracy. GP classification is then performed using this number of selected features.
*Procedure 5:* The specific GLM feature selection method identifies the 1000 most ‘active’ voxels in our experiment. RFA is then performed with a range of selected features from 5 to 150 [10], selecting the number of features leading to the best generalization accuracy. SVM classification is then performed using this number of selected features.

**Table 1:** Outline of the different combinations of features extraction and classification methods used in the present study.

	Features Extraction		Classification method	
	GLM	RFA	SVM	GP
**Procedure 1**	specific		X	
**Procedure 2**	specific			X
**Procedure 3**	global	X		X
**Procedure 4**	specific	X		X
**Procedure 5**	specific	X	X	

With procedures 1 and 2, features are only selected by a GLM analysis and accuracies were computed in terms of leave-one-block-out cross-validations: at each step, one block containing m data points (i.e. 27 consecutive images of faces, buildings and animals for the maze exploration session, and all the mentally represented images of 6 consecutive paths for the mental imagery session) was left out as a test set while the others were used as a training set to build the SVM or GP model. With procedures 3, 4 and 5, a double leave-one-block-out cross-validation was needed (Figure 2) to ensure the independence of feature extraction and classification [6], [24]. The inner cross-validation is used to determine the number of features to be selected by RFA and therefore obtain an optimal SVM model on n-1 blocks while the outer leave-one-block-out cross-validation tested the built SVM or GP model. The outer cross-validation is performed on the same folds for all procedures, which therefore allows their comparison.

To obtain one single accuracy measure for each subject (over all the categories instead of three measures, one for each binary comparison), an Error-Correcting Output Code approach (ECOC, [32]) was considered, inspired from [31]: each class was represented by a codeword of length n, n being the number of binary classifications. Each classifier votes for the two classes that it was built for, and for each class the votes of all the classifiers are assembled so as to constitute a ‘codeword’ for each class, which was further used for comparison with test points. For each test instance, the distance between the vector computed from the predictions of the set of classifiers and the correct codewords associated to each possible class can then be computed and the class characterized by the smallest distance from the predicted vector is selected. In this work, three classes were considered (i.e. faces, buildings and animals), leading to codewords of length three. In procedures 1 and 5, the votes were defined using the +1/−1 outputs of the SVM binary classifiers (table 2) and the final class of a test point was attributed according to the smallest Hamming distance between this vector and all the candidate class codewords [31]. In procedures 2, 3 and 4, the codewords were defined in terms of probabilities obtained from each GP binary classification (table 2), and the distance was computed as the sum of the differences between the table and the probabilities obtained from each binary classifier. The difference between the two tables, binary and probabilistic, lies in the precision of the distance measure between the vector of predictions associated to a test instance and the different codewords (see Appendix S1 for an illustration).

**Table 2:** Codewords using predictions (left part) and probabilities (right part) of the binary classifier.

	Codewords using predictions (SVM)			Codewords using probabilities (GP)		
	F-B	F-A	B-A	F-B	F-A	B-A
Faces	1	1	0	1	1	0.5
Buildings	−1	0	1	0	0.5	1
Animals	0	−1	−1	0.5	0	0

The lines correspond to the considered classes while the columns represent the different binary comparisons (F: faces, B: buildings and A: animals).

Since each test point is attributed to a class, measures of accuracy over categories can simply be computed as the total number of test instances correctly classified divided by the total number of test instances. Class accuracies can also be derived, calculated as the number of test instances which were correctly classified to the considered class, divided by the number of test instances from this class. However, due to class imbalances in the mental imagery session, balanced accuracy measures were computed (as the mean of the class accuracies) to take the different frequencies of the classes into account and therefore replaced the over categories accuracy measure in this work. To assess the significance of the classification of each procedure on each subject, permutations of the training set labels were performed (labels were permuted within each block to preserve class frequencies in each temporally correlated block). P-values were then associated to the balanced accuracy measure of each subject, by comparing it to the balanced accuracy obtained when shuffling the labels 100 times per cross-validation step (i.e. 1500 times for exploration and 900 times for mental imagery in total). Permutations suited best this within-subject framework since it avoided considering the CV folds (i.e. the blocks from a same subject and session) as independent and identically distributed [33].

Finally, the different procedures were compared using Friedman tests based on the balanced accuracy across subjects but also on the accuracies for each class, which allowed a better insight on the particularities of each modeling technique. In particular, the proportions of Support Vectors (SV) for each class was computed for the three SVM binary classifiers of procedure 5 to investigate the effect of an unbalanced data set on the SVM technique and establish their relation to class accuracies.

### Softwares

The SVM implementation used is the LIBSVM toolbox (Chang C. C. and Lin, C. J., http://www.csie.ntu.edu.tw/~cjlin/libsvm/) with a PROBID interface (Andre Marquand and Janaina Mourao-Miranda, http://www.brainmap.co.uk/). The choice of LIBSVM, which does not employ different weights for class errors, was driven by the wish to use SVM as a ‘reference’ method, and therefore led to the selection of a standard implementation of a classical SVM as is commonly employed in decoding neuroimaging data. The GP implementation used is the compiled version coded by C. E. Rasmussen and C. K. I. Williams (http://www.gaussianprocess.org/gpml/) and interfaced in PROBID.

## Results

### Behavioral data

During exploration, 135 events were extracted for each category, each one lasting 3 seconds. During mental imagery, the number of extracted events and their corresponding duration were variable depending on the volunteer's ability to retrieve the different images forming the requested mental path (Table 3). A Friedman test showed an effect of category on the number of events (p = 0.0016). Post hoc paired Wilcoxon signed rank tests showed that the numbers of events in the faces and animals categories was significantly larger than in the buildings category (F-B: p = 0.0027, A-B: p = 0.0021, Bonferroni corrected for multiples comparison) whereas no significant difference was detected between faces and animals (p = 0.0877).

**Table 3:** Number of events of each category extracted from the mental imagery session and corresponding percentages, for each subject.

	Number of events in each category during mental imagery		
Subject index	Faces	Buildings	Animals
S1	69 (44.23%)	30 (19.23%)	57 (36.54%)
S2	52 (30.41%)	43 (25.15%)	76 (44.44%)
S3	76 (55.07%)	11 (07.97%)	51 (36.96%)
S4	47 (30.32%)	58 (37.42%)	50 (32.29%)
S5	63 (37.72%)	50 (29.94%)	54 (32.34%)
S6	74 (39.36%)	42 (22.34%)	72 (38.30%)
S7	65 (39.39%)	43 (26.06%)	57 (34.55%)
S8	70 (41.42%)	36 (21.30%)	63 (37.28%)
S9	43 (40.19%)	31 (28.97%)	33 (30.84%)
S10	67 (38.29%)	44 (25.14%)	64 (36.57%)
S11	18 (21.69%)	32 (38.55%)	33 (39.76%)
S12	37 (45.68%)	20 (24.69%)	24 (29.63%)
S13	69 (41.07%)	31 (18.45%)	68 (40.48%)
S14	51 (44.74%)	22 (19.30%)	41 (35.96%)
S15	55 (32.54%)	58 (34.32%)	56 (33.14%)
S16	77 (38.89%)	53 (26.77%)	68 (34.34%)
**Mean**	**58.31 (38.81%)**	**37.75 (25.35%)**	**54.18 (35.84%)**

The reference percentage is 33%, corresponding to three perfectly balanced classes.

These findings were consistent with the results of the memory test conducted outside the scanner (Table 4): the percentage of correct answers is significantly lower (Friedman test: p = 3.2*10^−6^, Post hoc Wilcoxon tests, p<0.05, Bonferroni corrected for multiple comparisons) for the buildings category than for the other categories. The absolute differences in performance between categories were the largest between faces and buildings and between animals and buildings. This result potentially affected the classification based on fRMI data as they rely on binary comparisons.

**Table 4:** Percentage of correct answers during the memory test session outside the scanner.

	% of correct answers:			Dif in %:		
Subject index	Faces	Buildings	Animals	F-B	F-A	B-A
S1	48.9	35.6	46.7	13.3	02.2	11.1
S2	55.6	11.1	28.9	44.4	26.7	17.8
S3	55.6	42.2	64.4	13.3	08.9	22.2
S4	75.6	53.3	68.9	22.2	06.7	15.6
S5	68.9	51.1	73.3	17.8	04.4	22.2
S6	62.2	31.1	46.7	31.1	15.6	15.6
S7	77.8	55.6	75.6	22.2	02.2	20.0
S8	77.8	42.2	68.9	35.6	08.9	26.7
S9	71.1	57.8	71.1	13.3	00.0	13.3
S10	51.1	22.2	51.1	28.9	00.0	28.9
S11	55.6	42.2	64.4	13.3	08.9	22.2
S12	53.3	08.9	35.6	44.4	17.8	26.7
S13	55.6	42.2	57.8	13.3	02.2	15.6
S14	40.0	15.6	48.9	24.4	08.9	33.3
S15	62.2	42.2	51.1	20.0	11.1	08.9
S16	64.4	42.2	46.7	22.2	17.8	04.4
**Mean**	**61.0**	**37.2**	**56.2**	**23.75**	**08.9**	**19.0**

The results are displayed in terms of percentage of correct answers for each category as well as the difference in percentage between categories. F-B = faces-buildings, F-A = faces-animals, B-A = buildings-animals.

However, no interaction could be detected betweent the performances of the subject and the number of events in each category (correlations: p>0.05).

### Feature extraction

The ‘global GLM feature selection’ option considered in procedure 3 led to about 35,000 selected voxels for both sessions (range: 30,372–40,527, mean: 36,203 for the exploration session; range: 29,996–38,541, mean: 34,172 for the mental imagery session).

The number of features extracted by RFA in procedures 3, 4 and 5 are summarized in Table 5, respectively. Procedure 3 identified 342.95 optimal features (302.11 for mental imagery, mean across blocks and across subjects) while procedures 4 and 5 selected 310.92 features (270.17 for mental imagery), the difference between procedures being significant (Friedman test, p<10^−4^) for exploration. Standard deviations in the number of voxels selected indicate a high variability across blocks for mental imagery, independently of the procedure. This high variability across blocks, precluding from any conclusion at the procedure level, is directly linked to the design of the session. For exploration, the variability across blocks is small for both procedures, suggesting that the computation of a GLM for each LOO-CV does not induce much variability in the subset of voxels selected (Friedman test on the residuals, p = 0.7276). Procedure 5 being identical to procedure 4 in terms of feature selection, Table 5 displays the sizes of the selected subsets of features for both procedures.

**Table 5:** Number of RFA selected features for procedures 3, 4 and 5.

	Procedure 3				Procedures 4 and 5			
	Exploration		Mental Imagery		Exploration		Mental Imagery	
Subject	Mean	Std	Mean	Std	Mean	Std	Mean	Std
S1	369	5	220	142	337	65	254	173
S2	225	100	256	205	307	91	218	176
S3	360	66	385	149	277	88	258	150
S4	369	3	191	134	274	76	272	185
S5	324	88	351	245	310	78	314	164
S6	341	89	357	274	326	67	297	163
S7	363	55	266	249	338	66	290	172
S8	375	4	349	262	365	29	278	204
S9	234	100	225	256	216	88	217	134
S10	354	89	346	153	314	103	306	190
S11	364	54	258	168	313	65	148	185
S12	352	58	509	201	366	28	304	222
S13	344	85	313	108	333	40	294	183
S14	353	51	295	237	282	83	341	212
S15	391	45	272	139	291	109	326	227
S16	370	30	241	201	327	67	205	165
**All**	**342.95**	**57.67**	**302.11**	**206.28**	**310.92**	**71.28**	**270.17**	**180.27**

The optimal subset of variables is represented for each subject by its average size (second and fourth columns) and standard deviation (third and fifth columns) across blocks for the exploration (second and third column) and mental imagery (fourth and fifth) sessions. The last line gives the mean and standard deviation across subjects. Results are presented in terms of mean and standard deviation across the number of features obtained after each cross-validation step.

For both sessions and all procedures, the selected voxels were mostly comprised in the ventral visual path (primary areas, Fusiform Face Area), parietal regions linked to spatial features and hippocampus related to navigation. Activation in these areas represented properly the different aspects of both tasks.

### Classification

**Figure 3:**
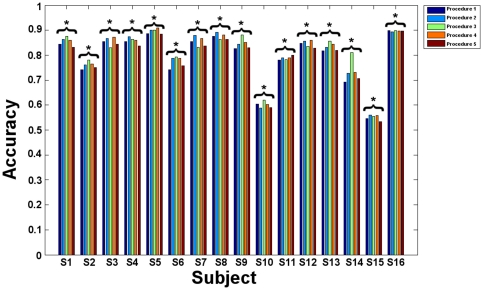
Exploration: Mean balanced accuracies obtained in the different procedures for all subjects. Procedure 1: specific GLM feature selection and SVM classification. Procedure 2: specific GLM feature selection and GP classification. Procedure 3: global GLM and RFA feature selections and GP classification. Procedure 4: specific GLM and RFA feature selections with GP classification. E Procedure 5: specific GLM and RFA feature selections with SVM classification. All results are significant.

**Figure 4:**
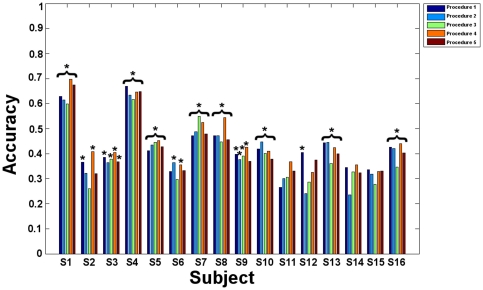
Mental imagery: Mean balanced accuracies obtained in the different procedures for all subjects. Procedure 1: specific GLM feature selection and SVM classification. Procedure 2: specific GLM feature selection and GP classification. Procedure 3: global GLM and RFA feature selections and GP classification. Procedure 4: specific GLM and RFA feature selections with GP classification. E Procedure 5: specific GLM and RFA feature selections with SVM classification. Significant classification accuracies are marked by stars *.

We first modeled the exploration session, before considering the mental imagery session. In the following sections, the results for both sessions and the four procedures are expressed for each category in terms of balanced accuracy (mean across blocks and significance for each subject, Figures 3–4).

#### Procedure 1

For exploration, the mean balanced accuracies were all above chance level, ranging from 54.57 to 89.88% (p<0.05). For mental imagery, mean balanced accuracies ranged from 26.59 to 67.01%. Low accuracy measures led to non significant results for subjects S6, S11, S14 (p = 0.07) and S15 (p>0.05).

#### Procedure 2

For exploration, GP classification provided mean balanced accuracies ranging from 56.05 to 90.12% (p<0.05). For mental imagery, mean balanced accuracies were comprised between 24.09 and 63.48%. The classification was not significant for subjects S2, S3 (p = 0.07), S11, S12, S14 and S15 (p>0.05).

#### Procedure 3

For exploration, mean balanced accuracies obtained using the RFA feature selection ranged from 55.56 to 90.12% (p<0.05). For mental imagery, mean balanced accuracies ranged from 25.94 to 61.65%. These results were not significant for subjects S2, S6, S11, S12, S14 and S15 (p>0.05).

#### Procedure 4

For exploration, the optimal subsets of features defined by GLM and RFA were associated with mean balanced accuracies ranging from 55.80 to 90.86% (p<0.05). For mental imagery, mean balanced ranged from 32.98 to 69.78%. However, non significant results were found for subjects S11, S12, S14 and S15 (p>0.05).

#### Procedure 5

For exploration, the optimal subsets of features defined by GLM and RFA were associated with mean balanced accuracies ranging from 53.33 to 89.63% (p<0.05). For mental imagery, mean balanced accuracies ranged from 32.05 to 67.50%, leading to non significant results for subjects S2, S6, S9 (p = 0.07), S11, S12 (p = 0.06), S14 and S15 (p = 0.06).

Overall mean balanced accuracies for the exploration session were significantly above chance for all the subjects and all procedures. For the mental imagery sessions, mean balanced accuracies were not significant for some subjects and some procedures: S2 (procedures 2, 3 and 5), S3 (procedure 2), S6 (procedures 1, 3 and 5), S9 (procedure 5), S11 (all procedures), S12 (procedures 2, 3, 4 and 5), S14 (all procedures) and S15 (all procedures).

### Comparison of procedures

#### Balanced accuracy

**Figure 5:**
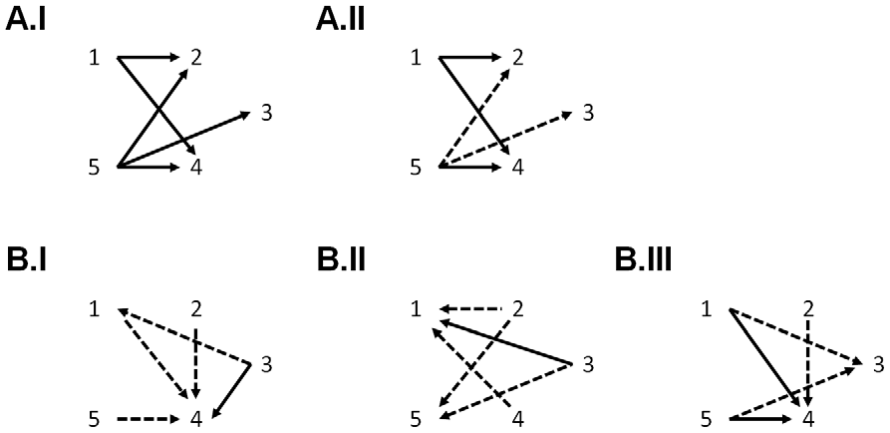
Schematic comparisons between procedures. The full arrows represent a significant difference (p<0.05, survives Bonferroni correction multiple comparisons) in performance between the two procedures linked, the arrow pointing to the best. The dashed arrows represent trends (p<0.05, but does not survive Bonferroni correction). A.I Exploration: differences in balanced accuracy. Procedures 2, 3 and 4 (GP) perform best. A.II Exploration: differences in animals class accuracy. B.I. Mental imagery: differences in balanced accuracy. Procedure 4 tended to perform best. B.II. Mental imagery: differences in faces class accuracy. Procedures 1 and 5 (SVM) tended to perform best. B.III. Mental imagery: differences in buildings class accuracy. Procedures 3 and 4 (tended to) perform best.

For exploration, the Friedman test on the over categories accuracy measures revealed significant differences (p<10^−4^) between procedures. Paired Wilcoxon signed rank tests showed that procedures 1 and 5 (SVM classification) performed significantly worse than procedures 2, 3 and 4 (p<0.05, Bonferroni corrected for multiple comparisons, Figure 5.A.I).

Similarly, for mental imagery, a Friedman test on the balanced accuracies showed a significant effect of procedure (p = 0.0012). The paired Wilcoxon signed rank tests showed that procedure 4 performed significantly better than procedure 3 (p = 7.76*10^−4^) and better than all other procedures (p<0.05, but does not survive Bonferroni correction for multiple comparisons). Procedure 3 also tended to perform worse than procedure 1 (p = 0.0437, not significant after Bonferroni correction, Figure 5.B.I).

#### Class accuracy

For exploration, there was an effect of procedure on the class accuracy measures only for the animal category (F: p = 0.0672, B: p = 0.1594 and A: p = 0.0017). Paired Wilcoxon signed rank tests showed that procedures 1 and 5 tended to perform worse than procedures 2, 3 and 4 for the animal category (p<0.05, corrected for multiple comparisons using Bonferroni correction, Figure 5.A.II).

For mental imagery, Friedman tests showed a significant effect of procedure on the classification of faces and buildings (p<10^−3^). Paired Wilcoxon signed rank tests on the class accuracy for faces showed that procedure 1 performed significantly better than procedure 3 (p = 0.0027), and better than procedures 2 and 4 (p<0.05, does not survive Bonferroni correction, Figure 5.B.II). Trends also indicated that procedure 5 led to higher accuracies than procedures 2 and 3 (2–5: p = 0.0295, 3–5: p = 0.0085, do not survive Bonferroni correction). The paired Wilcoxon signed rank tests on the class accuracy for buildings showed that procedure 4 performed significantly better than procedures 1, 3 and 5 (p<0.05, Bonferroni corrected for multiple comparisons). Trends showing better performance of procedure 3 over procedures 1 and 5, and of procedure 4 over procedure 2 were also noticed but not significant (1–3: p = 0.0327, 3–5: p = 0.0166, 2–4: p = 0.0131 do not survive the Bonferroni correction, Figure 5.B.III). No other significant differences in class accuracies were noted.

**Figure 6:**
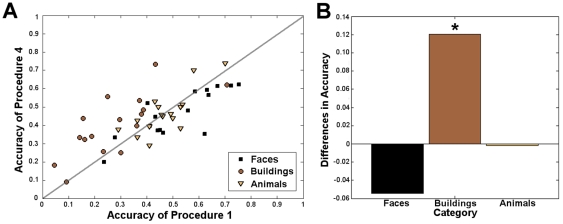
Class accuracies obtained by the procedures 1 and 4 for all subjects. The faces category is represented in black, the buildings category, in dark brown, and the animals category, in light brown. A Comparison of the accuracy values. x-axis: class accuracies obtained using procedure 1. y-axis: class accuracies obtained using procedure 4. Most points corresponding to class accuracies of buildings (represented by dark brown circles) are above the 45° line, meaning that the buildings were better classified using procedure 4. B Average difference in class accuracy between procedures 1 and 4. This figure shows that the difference in buildings classification is significant across subjects, while this is not the case for the faces and animals categories.

This result is illustrated in Figure 6, comparing the class accuracies obtained for each subject with procedures 1 and 4 (Figure 6.A). It was observed that procedure 4 performed always better than procedure 1 to classify buildings. Figure 6.B assessed the significance of this difference in performance between procedure 1 and procedure 4. Only the building classification was significantly different, i.e. worse for procedure 1 compared to procedure 4. Similar results were obtained when comparing procedures 4 and 5 in terms of buildings accuracy (not shown).

#### Support Vectors proportion

Procedures 1 and 5 showing no significant difference in balanced or class accuracies, support vectors (SV) proportions were computed from each SVM binary classifier of procedure 5 (percentage of faces SV for the F-B and F-A comparisons and percentage of animals SV for the B-A comparison).

For exploration, a significant effect of the binary classifier on the proportions of SV was assessed (p<0.05): post hoc Wilcoxon tests revealed that the proportion of SV in the faces category (F-B classifier) was significantly higher than in the faces category for the F-A classifier and in the animals category for the B-A classifier (p<0.05, Bonferroni corrected). Whilst the proportions of faces SV in the F-B and F-A classifiers differed significantly from 50% (F-B>50%: p = 0.0084, F-A<50%: p = 0.0045), no significant correlation could be found between the SV proportions and the class accuracies (0.0735<p<0.8309). Moreover, the sign of some correlation coefficients were not consistent with the expected effect on class accuracy (for example, a positive correlation was found between the proportion of faces SV and the class accuracy of buildings, while one would expect the class accuracy of buildings to decrease when increasing the proportion of faces SV).

For mental imagery, a Friedman test also showed an effect of the binary classifier on the proportions of SV (p = 6.81*10^−4^). Post hoc Wilcoxon signed rank tests revealed that the proportions of SV in the faces (for the F-B classifier) and in the animals (for the B-A classifier) categories were significantly higher than in the faces category for the F-A classifier (p<0.05, Bonferroni corrected). SV proportions in the faces (F-B) and animals (B-A) categories were significantly higher than 50% (faces in F-B: p = 0.0151 and animals in B-A: p = 0.0013). Significant anti-correlations were found between the class accuracy of buildings and the proportion of faces SV in the F-B classifier (p = 0.0145), and the proportion of animals SV in the B-A classifier (p = 0.0190). Although no other significant correlation was assessed between class accuracy and SV proportions, the signs of all correlation coefficients were consistent with the expected effects.

### Effect of behavioural data

#### Number of events

When considering the classification of mental imagery using all procedures (i.e. accuracies have been averaged across procedures), a significant effect of the number of events could be detected for the faces and buildings category (correlations, faces: rho = 0.5341, p = 0.0331, buildings: rho = 0.5117, p = 0.0428). When investigating the procedures individually, significant correlations were found between the number of faces events and the classification of faces in procedures 1 and 5 (SVM classifier). All procedures showed a significant correlation between the number of buildings events and the classification of images of that category (p<0.05).

#### Behavioural performances

No significant correlations were found between the accuracy of all classifiers and the performance of the subjects at the test session led outside the scanner. However, for mental imagery, trends still indicated an effect of the total number of correct answers on the balanced accuracy (p = 0.0867) as well as a correlation between the classification of buildings and the number of correct answers in the buildings category (p = 0.0575). In particular, procedures 2 and 3 (resp. 3, 4 and 5) showed significant correlation between the subjects' performances and the balanced accuracy over the three categories (resp. for the buildings category). The other procedures still showed trends, but the correlations were not significant (over categories: p(P1) = 0.1367, p(P4) = 0.0560, p(P5) = 0.092, buildings: p(P1) = 0.0509, p(P2) = 0.0686).

## Discussion

We tested the performance of different classification procedures on two separate fMRI time series. Whereas the experimental design of the exploration session imposed a paced and regular succession of stimulus categories, the mental imagery session was characterized by imbalanced numbers of trials between categories, a self-paced succession of individual trials and subject related task performance. The uneven number of events across categories was related to the disparity in individual memory performance, pictures of buildings being significantly less well remembered than the two other classes of stimuli. In addition, the succession of events of variable durations, sometimes beyond temporal resolution of fMRI, put a further strain on classification procedures. The best combinations of techniques (namely procedures 1 and 4) were able to classify accurately, i.e. significantly above chance level, the mental images from 12 out of the 16 subjects.

### Methodological implications

When classifying the well-controlled session, results indicated that SVM performed significantly worse than GP. No effect of the feature selection (either specific GLM, global GLM and RFA or specific GLM and RFA) could be detected, which does not correspond to what was reported in the literature [6], [27]–[29]. This result indicates that for this well-controlled experiment, using a GLM filter or a RFA embedded wrapper leads to the same performances.

However, when considering the mental imagery session, performance of the considered procedures indicates that GP might be more sensitive to the addition of irrelevant features than SVM. This hypothesis is supported by the fact that a univariate feature extraction by a specific GLM substantially improved classification accuracy. Indeed, procedure 4 (specific GLM-RFA-GP) achieved better accuracies than procedure 3 (global GLM-RFA-GP), which needed more computational time for significantly poorer results. While the authors of [27] suggested that such a univariate feature selection step ‘may improve’ the accuracy of intrasubject classification, we show that this improvement is significant for the considered GLM contrasts and data sets. In addition, combining a specific GLM with a second multivariate step further improved feature selection, as indicated by the higher accuracy achieved by procedure 4 relative to 2 (specific GLM-GP). This result is in agreement with [6], [28] and [29], which stated that the combination of a univariate selection of ‘active’ voxels combined to a multivariate selection of ‘discriminant’ voxels led to the best performances of classifiers. Since the main difference between the two classification techniques considered lies in the sparseness of SVM [14], one plausible explanation for this result could be that GP overfits the data by using all the data points to define the separating function. This overfitting can then be reduced via a two-level feature selection approach. However, procedures 1 and 5 (specific GLM-RFA-SVM) showed similar performances, suggesting that the RFA step did not bring further relevant information to the SVM classifier. Furthermore, it allowed higher accuracies to be reached when permuting the labels, which led to non-significant results for 7 subjects out of 16.

Once the optimal subset of features was defined, the performances of GP and SVM classifiers showed only slight differences (trend that procedure 4 performs better but not significant). However, GP seems more robust than SVM for classifying imbalanced data sets, as the former achieved a significantly better accuracy for the least represented class (i.e., buildings in the current study). This result might be explained by the sparseness of SVM since significantly different proportions of support vectors between the binary classifiers, which correlated with the obtained class accuracies, were revealed.

### Functional significance of classification results

The two behavioural measures computed in this work (i.e. the number of events in each category and the performances to a memory test led outside the scanner) correlated significantly with the performances of the procedures, especially for the least represented class. The small number of observations precludes any definitive conclusion. However, these findings suggest that the ability to reinstate category-specific activity patterns within specific occipito-temporal areas supports memory retrieval.

### Conclusions

The results show that for fMRI time series which include complex, unbalanced self-generated mental states, best accuracies are obtained by a feature selection combining a specific GLM and a recursive feature addition. Whilst the advantage of GP over SVM to classifying this type of data is small (in terms of balanced accuracy), the former seems more appropriate for markedly imbalanced data sets, and thus preferable for more realistic experimental setups.

## Supporting Information

Appendix S1 Outputs of SVM and GP classifiers (second and third lines respectively) applied on one example data point.
(DOCX)
