# Supplementary material for: Decoding Semi-Constrained Brain Activity from fMRI Using Support Vector Machines and Gaussian Processes
Source: PLoS One. 2012 Apr 26;7(4):e35860. doi: 10.1371/journal.pone.0035860 (PMC3338538; doi:10.1371/journal.pone.0035860)
Supplement: Appendix S1 — Outputs of SVM and GP classifiers (second and third lines respectively) applied on one example data point. (DOCX) [file pone.0035860.s001.docx]

Appendix S1

To illustrate the difference between the +1/-1 or probability based codewords, let’s consider an example (table S1). When using the SVM predictions, the distance measure used to assign the final class is the Hamming distance (i.e. the number of differing bits), which would give for the three classes:

L^F^ = 1 + 1 + 1 =3

L^B^ = 0 + 1 + 1 =2

L^A^ = 1 + 0 + 0 =1

where L represents the final score of each class. In the present case, the class ‘animals’ is assigned to the test point since it shows the smallest final score L, which leads to a misclassification.

On the other hand, the probability based codewords lead to the following scores:

L^F^ = |1 - 0.2| + |1 – 0.3| + |0.5 – 0.5| =1.5

L^B^ = |0 - 0.2| + |0.5 – 0.3| + |1 – 0.5| =0.9

L^A^ = |0.5 - 0.2| + |0 – 0.3| + |0 – 0.5| =1.1

The ‘building’ class was correctly assigned to the test point. Therefore, whenever possible (i.e. when the classifier returned probabilities), the probability based codewords were used to perform the ECOC scheme.

**Table S1**

| Test point  (true class: Building) | F-B | F-A | B-A |
| --- | --- | --- | --- |
| SVM predictions | -1 | -1 | -1 |
| GP probabilities | 0.2 | 0.3 | 0.5 |
